# Adult attachment and intimate relationship satisfaction among university students: the chain mediating roles of appreciation and sense of giving

**DOI:** 10.3389/fpsyt.2026.1758775

**Published:** 2026-04-24

**Authors:** Mengyuan Zhu, Jiang Tan, Zuli Zheng, Lifan Liang, Qing Li, Juanrong Wen, Zhixia Wang, Aimei Zhang, Gang Wu, Jiaoying Liu, Yanping Shu

**Affiliations:** 1 School of Psychology, Guizhou Normal University, Guiyang, China; 2 Department of Women and Child Psychiatry, The Second People’s Hospital of Guizhou, Guiyang, China; 3 School of Digital Economy and Finance, Guizhou University of Commerce, Guiyang, China; 4 The Second Clinical Medical College, Guizhou University of Traditional Chinese Medicine, Guiyang, China; 5 The First Clinical Medical College, Zunyi Medical University, Guiyang, China; 6 College of Medical Humanities, Guizhou Medical University, Guiyang, China

**Keywords:** adult attachment, appreciation, chain mediation, relationship satisfaction, sense of giving, young adults

## Abstract

**Background:**

Satisfying intimate relationships are fundamental to young adults’ psychological well-being. Although adult attachment theory provides a robust framework for understanding relationship quality, the mechanisms linking higher attachment anxiety and avoidance to lower relationship satisfaction remain underexplored. This study tested a chain mediation model in which appreciation (both expressed and felt) and sense of giving sequentially mediate the link between insecure attachment and relationship satisfaction.

**Methods:**

A cross-sectional survey was conducted with 536 university students (mean age = 21.67 years; 55.8% female) currently in romantic relationships. Participants completed validated self-report questionnaires assessing higher attachment avoidance and anxiety, appreciation (appreciating one’s partner and feeling appreciated), sense of giving, and relationship satisfaction. Chain mediation analyses were performed using the SPSS PROCESS macro with 5,000 bootstrap resamples to evaluate the significance of indirect effects.

**Results:**

Attachment significantly and negatively predicted relationship satisfaction. In the first chain mediation model, higher levels of both attachment avoidance and anxiety were negatively associated with appreciation of one’s partner. Appreciation, in turn, was positively associated with a greater sense of giving, which was subsequently linked to higher relationship satisfaction. After controlling for demographic and relational covariates, the key findings remained robust. The sequential indirect effect (Attachment → Appreciating → Giving → Satisfaction) was significant for both higher attachment avoidance (effect = -0.17, 95% *CI* [-0.22, -0.14]) and anxiety (effect = -0.07, 95% *CI* [-0.10, -0.03]). Similarly, the sequential indirect effect through feeling appreciated (Attachment → Feeling Appreciated → Giving → Satisfaction) was significant for both higher avoidance (effect = -0.10, 95% *CI* [-0.14, -0.07]) and anxiety (effect = -0.09, 95% *CI* [-0.12, -0.06]).

**Conclusion:**

Insecure attachment was negatively associated with intimate relationship satisfaction through a sequential pathway involving appreciation and sense of giving. These cross-sectional findings suggest that appreciation processes may represent a promising intervention target. Clinical or preventive strategies enhancing the expression and perception of appreciation could potentially improve relationship satisfaction among young adults with insecure attachment orientations.

## 1 Introduction

Intimate relationships constitute a fundamental cornerstone of psychological well-being, particularly during emerging adulthood (ages 18-25), when individuals navigate identity formation and interpersonal intimacy (1, 2). For university students, the quality of romantic relationships carries profound implications across multiple domains. Satisfying intimate relationships robustly predict enhanced psychological adjustment, with longitudinal studies revealing that relationship quality during college years forecasts mental health trajectories into adulthood (3). High-quality romantic relationships are also associated with greater academic engagement, improved stress resilience, and enhanced life satisfaction (4, 5). Conversely, relationship distress emerges as a potent risk factor for depressive symptoms, anxiety disorders, and diminished self-worth (4). Recent epidemiological data indicate that relationship distress is a primary reason college students seek counseling services (6). Relationship satisfaction—individuals’ subjective global evaluation of their romantic partnership—serves as a crucial indicator of relationship quality and stability (7). Given these far-reaching consequences, elucidating the mechanisms associated with relationship satisfaction in young adults holds significant theoretical and practical value.

Attachment theory provides a comprehensive framework for understanding individual differences in relationship functioning. According to this perspective, early caregiving experiences shape internal working models—cognitive-affective schemas that guide expectations, emotions, and behaviors within close relationships (6, 8, 9). Contemporary research conceptualizes adult attachment along two orthogonal dimensions: attachment anxiety and attachment avoidance (9, 10). Individuals high in attachment anxiety harbor persistent fears of abandonment and rejection, exhibiting hyperactivating strategies characterized by excessive reassurance-seeking and emotional dysregulation (11–14). In contrast, individuals high in attachment avoidance maintain defensive self-reliance and employ deactivating strategies involving emotional distancing and intimacy avoidance (11, 15, 16). Meta-analytic evidence confirms that both dimensions are negatively associated with relationship satisfaction (17). The excessive dependency needs of individuals high in attachment anxiety often contribute to negative interaction cycles, while the emotional unavailability of individuals high in attachment avoidance impedes the deep connection essential for relationship fulfillment (16, 18, 19).

Although the direct association between insecure attachment and relationship dissatisfaction is well established, the specific mechanisms linking attachment orientations to relationship outcomes remain incompletely understood (20). The present study addresses this gap by proposing a chain mediation model in which appreciation and sense of giving sequentially mediate the attachment–satisfaction link. Appreciation represents a dyadic construct encompassing both expressing appreciation toward one’s partner and perceiving appreciation from one’s partner (21, 22). Recent work distinguishes appreciation from gratitude, highlighting that appreciation focuses on recognizing partners’ intrinsic qualities rather than specific behaviors (23). This distinction proves important, as appreciation taps into deeper validation of the partner’s core self. The experience of feeling appreciated fulfills fundamental psychological needs for competence, autonomy, and relatedness (24). Attachment theory generates compelling predictions regarding appreciation deficits among individuals with higher attachment insecurity (6, 19, 25). The discomfort with emotional expression in individuals high in attachment avoidance may reduce their capacity to express appreciation, as doing so requires acknowledging dependence (26). Similarly, the negative self-models in individuals high in attachment anxiety may lead them to discount partners’ appreciative gestures (27, 28). Longitudinal studies demonstrate that perceived appreciation functions as a critical resource for relationship maintenance (29, 30).

The sense of giving represents individuals’ subjective appraisal of their contributions within romantic relationships, distinct from objective behavioral frequencies (7). This construct captures how individuals experience their relationship investments—whether as meaningful expressions of love or as burdensome obligations (31). Contemporary social exchange perspectives emphasize that relationship satisfaction depends not merely on the amount of giving but on individuals’ subjective experience of their contributions (31). When individuals perceive their giving as meaningful investments, such contributions are associated with enhanced well-being through an increased sense of purpose and connection (22, 32). However, when giving is experienced as unreciprocated or obligatory, it may deplete psychological resources and reduce satisfaction (33). Research reveals that partners’ perceptions of giving asymmetry more strongly predict relationship outcomes than actual behavioral disparities (34, 35). Moreover, the sense of giving appears particularly sensitive to partner responsiveness; when partners respond appreciatively, givers experience enhanced intrinsic motivation (32, 34, 36, 37).

The primary theoretical innovation of our model lies in proposing a sequential pathway wherein appreciation precedes and shapes the sense of giving. We posit that appreciation acts as a foundational mechanism that reframes an individual’s appraisal of their relational contributions (21, 38–41). Grounded in self-determination theory, this sequence suggests that feeling appreciated fulfills core psychological needs for relatedness, autonomy, and competence (42, 43). This need-satisfaction fosters autonomous motivation for relationship investments. When appreciation is present, giving is subjectively experienced as a meaningful and intrinsically rewarding act of commitment (44, 45). Conversely, when appreciation is absent—a common downstream correlate of insecure attachment—the same contributions may be appraised as unreciprocated costs, leading to resentment and eroded satisfaction (33, 46). This theoretically-derived sequence integrates attachment theory, which explains the initial disruption in appreciation processes (47, 48), with motivational principles that clarify how appreciation subsequently alters the psychological meaning of giving (49). Based on this framework, we propose the following hypotheses:

**H1:** Higher attachment avoidance and higher attachment anxiety will each be negatively associated with intimate relationship satisfaction.
**H2:** Appreciation (both appreciating one’s partner and feeling appreciated) will negatively mediate the association between insecure attachment dimensions and intimate relationship satisfaction. Specifically, we predict that higher insecure attachment will be associated with lower levels of appreciation, which in turn will predict lower relationship satisfaction, resulting in a negative indirect effect.
**H3:** Sense of giving will negatively mediate the association between insecure attachment dimensions and intimate relationship satisfaction. We expect that higher insecure attachment will be associated with a diminished sense of giving, which will subsequently predict lower relationship satisfaction, yielding a negative indirect effect.
**H4:** A sequential mediation model will be supported, wherein the association between insecure attachment dimensions and intimate relationship satisfaction is explained by a negative indirect pathway through appreciation and subsequently sense of giving. Specifically, we hypothesize that higher insecure attachment will be associated with lower appreciation, which in turn will be linked to a lower sense of giving, ultimately predicting lower relationship satisfaction.

## 2 Methods

### 2.1 Participants and procedure

This study employed a convenience sampling strategy to recruit undergraduate and graduate students from multiple universities in China. Data were collected between June 2025 and November 2025. Recruitment occurred through announcements in university classes, posts on student social media groups, and direct on-site invitations in university libraries and study rooms. Although the survey was administered online using the Wenjuanxing (www.wjx.cn) and TC Lab (www.testcloudlab.com) platforms, a substantial portion of participants completed the questionnaire in these semi-supervised settings. This context, combined with a concise instrument design, likely contributed to focused participant engagement and reduced variability in completion times.

The inclusion criteria for participation were (1): being a currently enrolled university or graduate student; (2) being in a heterosexual romantic relationship; and (3) having a relationship duration of at least three months. An initial sample of 600 students was invited to complete the online questionnaire, which took an average of 223 seconds (*SD* = 16.97) to complete. From this initial pool, 64 responses were excluded based on pre-defined data quality checks. Specifically, we removed participants who: (1) indicated they were not currently in a romantic relationship; (2) reported a student status other than university or graduate student; (3) exhibited invariant or patterned responding (e.g., selecting the same answer for all items); or (4) had completion times that fell more than three standard deviations from the mean. This screening process yielded a final valid sample of 536 participants, representing an effective response rate of 89.33%.

The sample comprised 299 females (55.8%) and 237 males (44.2%). Regarding family structure, 197 participants (36.8%) were only children, and 339 (63.2%) had siblings. The duration of romantic relationships was distributed as follows: 0–6 months (*N* = 144, 26.9%), 6 months to 1 year (*N* = 133, 24.8%), 1–3 years (*N* = 172, 32.1%), and over 3 years (*N* = 87, 16.2%). Participants ranged in age from 18 to 27 years, with a mean age of 21.67 years (*SD* = 2.20).

### 2.2 Measures

#### 2.2.1 Attachment dimensions

Attachment dimensions were assessed using a 7-item Chinese version of the Experiences in Close Relationships–Relationship Structures questionnaire (ECR-RS). This instrument is an adaptation of the original scale developed by Fraley et al. (49), which was revised and validated for Chinese populations by Peng et al. (50). The adapted version omitted two items from the original 9-item scale. This modification was based on their finding that these two items demonstrated low factor loadings in validation analyses with Chinese student samples. The authors attributed this to potential cultural differences in the expression and interpretation of certain attachment-related behaviors, suggesting the excluded items might not hold the same conceptual meaning in a Chinese context. The resulting 7-item version demonstrated robust psychometric properties and is considered more culturally appropriate for assessing attachment in China.

This 7-item instrument measures two dimensions: attachment avoidance (4 items, e.g., “I am not comfortable opening up to my romantic partner”) and attachment anxiety (3 items, e.g., “I worry that my romantic partner might abandon me”). Items 1–3 were reverse-scored. Participants rated each item on a 7-point Likert scale ranging from 1 (not at all true) to 7 (very true). Higher subscale scores indicate greater levels of attachment avoidance or anxiety. In this study, *Cronbach’s α* coefficients were 0.69 for attachment avoidance and 0.84 for attachment anxiety.

#### 2.2.2 Appreciation

Appreciation was measured using the Chinese version of the Appreciation in Relationships Scale, developed by Gordon et al. (51) and adapted by Zhang et al. (52). This 13-item scale assesses two components: appreciating one’s partner (7 items) and feeling appreciated by one’s partner (6 items). Responses were recorded on a 7-point Likert scale from 1 (strongly disagree) to 7 (strongly agree). Items 4 and 12 were reverse-scored. Higher scores reflect greater appreciation for one’s partner or greater perceived appreciation from one’s partner. *Cronbach’s α* coefficients were 0.79 for the appreciating subscale and 0.77 for the feeling appreciated subscale.

#### 2.2.3 Sense of giving

Sense of giving was assessed using the Sense of Giving Questionnaire, developed by Jiang et al. (53). This 14-item instrument comprises three subscales: cognitive giving(e.g., “I consider my partner as part of my future plans”), emotional giving(e.g., “I provide my partner with ongoing care when he/she is in trouble”), and behavioral giving(e.g., “I would do things I don’t want to do for my partner”). All items were rated on a 7-point Likert scale anchored at 1 (not at all true) and 7 (very true). Item 7 was reverse-scored. Higher total scores signify a greater perceived sense of one’s own contributions to the relationship. *Cronbach’s α* coefficients were 0.70, 0.66, and 0.77 for cognitive, emotional, and behavioral giving, respectively; the total scale α was 0.86.

#### 2.2.4 Intimate relationship satisfaction

Intimate relationship satisfaction was measured using the 6-item short form of the Quality of Relationship Inventory, adapted by Patrick et al. (54). Participants responded to each item on a 7-point Likert scale ranging from 1 (not at all true) to 7 (very true). Higher scores indicate greater relationship satisfaction. The scale demonstrated excellent internal consistency (*Cronbach’s α* = 0.89).

### 2.3 Data analysis

Data were entered and organized using Microsoft Excel 2019. All statistical analyses were conducted using IBM SPSS Statistics (Version 26.0). Descriptive statistics and Pearson correlations were computed to characterize the sample and examine relationships among the study variables.

To test the hypothesized chain mediation model, we employed the PROCESS macro (Version 3.3) for SPSS developed by Hayes (55). Specifically, Model 6 was selected to examine the proposed serial mediation pathways. This model estimates a three-path mediated effect (i.e., X → M1 → M2 → Y), allowing for the simultaneous assessment of specific indirect effects through each mediator individually and through the sequential chain of both mediators. In our analyses, adult attachment dimensions (attachment avoidance and attachment anxiety) served as the independent variables (X). Appreciation (operationalized separately as appreciating one’s partner and feeling appreciated by one’s partner) served as the first mediator (M1), and sense of giving served as the second mediator (M2). Intimate relationship satisfaction was the dependent variable (Y). Four separate models were estimated: two models examined the pathway through appreciating one’s partner (one for attachment avoidance and one for attachment anxiety), and two models examined the pathway through feeling appreciated by one’s partner.

The total effect of the independent variable on the dependent variable was decomposed into direct and indirect components. The direct effect represents the association between attachment and relationship satisfaction after accounting for the mediators. The total indirect effect represents the sum of all mediated pathways. Specific indirect effects were calculated for three pathways (1): the indirect effect through appreciation only (X → M1 → Y); (2) the indirect effect through sense of giving only (X → M2 → Y); and (3) the sequential indirect effect through both mediators in the hypothesized order (X → M1 → M2 → Y). The significance of all indirect effects was evaluated using a bias-corrected bootstrapping procedure with 5,000 resamples (56). An indirect effect was considered statistically significant if its 95% bias-corrected confidence interval did not include zero. This bootstrapping approach is recommended for mediation analysis because it does not assume normality of the sampling distribution of indirect effects and provides more accurate Type I error rates and statistical power compared to traditional methods such as the Sobel test (57).

To test the robustness of our findings and control for potential confounding variables, we included gender, age, only-child status, long-distance relationship status, and relationship duration as covariates in all mediation models (7, 58–62). These covariates were selected based on prior literature indicating their potential associations with attachment orientations and relationship outcomes.

## 3 Results

### 3.1 Common method variance analysis

Because all data were collected via self-report measures, Harman’s single-factor test was conducted to assess potential common method variance (CMV). An unrotated exploratory factor analysis extracted eight factors with eigenvalues greater than 1. The first factor accounted for 30.67% of the total variance, below the recommended 40% threshold, suggesting that CMV did not pose a substantial concern in this study.

### 3.2 Descriptive statistics and correlation analysis

Descriptive statistics and Pearson correlation coefficients for all study variables are presented in **Table 1**; **Supplementary Figure 1**. Regarding demographic and relational covariates, gender was significantly correlated with only-child status (*r* = 0.18, *p* <.01), sense of giving (*r* = -0.30, *p* <.01), and intimate relationship satisfaction (*r* = -0.16, *p* <.01). Age was positively associated with relationship duration (*r* = 0.23, *p* <.05). Relationship duration showed significant positive correlations with sense of giving (*r* = 0.15, *p* <.01) and intimate relationship satisfaction (*r* = 0.21, *p* <.01), and a significant negative correlation with higher attachment avoidance (*r* = -0.20, *p* <.01). After controlling for demographic variables, both higher attachment avoidance and higher attachment anxiety were significantly and negatively correlated with all four relational variables. Specifically, higher attachment avoidance showed moderate to strong negative associations with appreciating one’s partner (*r* = -0.45, *p* <.01), feeling appreciated by partner (*r* = -0.36, *p* <.01), sense of giving *(r* = -0.31, *p* <.01), and intimate relationship satisfaction (*r* = -0.47, *p* <.01). Higher attachment anxiety demonstrated a similar pattern, with significant negative correlations with appreciating one’s partner (*r* = -0.21, *p* <.01), feeling appreciated by partner (*r* = -0.32, *p* <.01), sense of giving (r = -0.14, *p* <.01), and intimate relationship satisfaction (*r* = -0.24, *p* <.01). The two attachment dimensions were positively intercorrelated (*r* = 0.31, *p* <.01).

**Table 1:** Descriptive statistics and bivariate correlations among study variables (*N* = 536).

Variable	*M*	*SD*	1	2	3	4	5	6	7	8	9	10	11
1 Gender	1.56	0.50	—										
2 Age	21.67	2.20	0.01	—									
3 Only-child status	1.63	0.48	0.18**	0.04	—								
4 Long-distance relationship status	1.50	0.50	-0.17**	0.09*	-0.11**	—							
5 Relationship duration	2.38	1.05	-0.01	0.23*	0.08	-0.16**	—						
6 Higher attachment avoidance	9.36	3.89	-0.07	-0.11*	0.03	-0.02	-0.20**	—					
7 Higher attachment anxiety	10.37	4.71	0.01	-0.10*	0.07	-0.13**	-0.13**	0.31**	—				
8 Appreciating one’s partner	39.44	5.76	-0.13**	0.06	-0.02	0.08	0.05	-0.45**	-0.21**	—			
9 Appreciated by partner	31.91	5.62	-0.12**	0.03	-0.08	0.13**	0.04	-0.36**	-0.32**	0.63**	—		
10 Sense of giving	76.76	10.84	-0.30**	0.03	-0.04	0.11**	0.15**	-0.31**	-0.14**	0.65**	0.54**	—	
11 Intimate relationship satisfaction	35.28	5.44	-0.16**	0.21	-0.05	0.12**	0.21**	-0.47**	-0.24**	0.59**	0.59**	0.66**	—

*M* represents the mean, *SD* represents the standard deviation. *p < 0.05, **p < 0.01.

Among the proposed mediators and outcome variable, all associations were positive and significant. Appreciating one’s partner was strongly correlated with feeling appreciated by partner (*r* = 0.63, *p* <.01), sense of giving (*r* = 0.65, *p* <.01), and intimate relationship satisfaction (*r* = 0.59, *p* <.01). Feeling appreciated by partner was also positively associated with sense of giving (*r* = 0.54, *p* <.01) and intimate relationship satisfaction (*r* = 0.59, *p* <.01). Sense of giving demonstrated a strong positive correlation with intimate relationship satisfaction (*r* = 0.66, *p* <.01). These correlation patterns provide preliminary support for the hypothesized relationships and justify subsequent mediation analyses.

### 3.3 Chain mediation model 1: Appreciating one’s partner and sense of giving

A chain mediation analysis was conducted to test whether appreciating one’s partner and sense of giving sequentially mediated the relationship between adult attachment dimensions and intimate relationship satisfaction.

**Figure 1:**
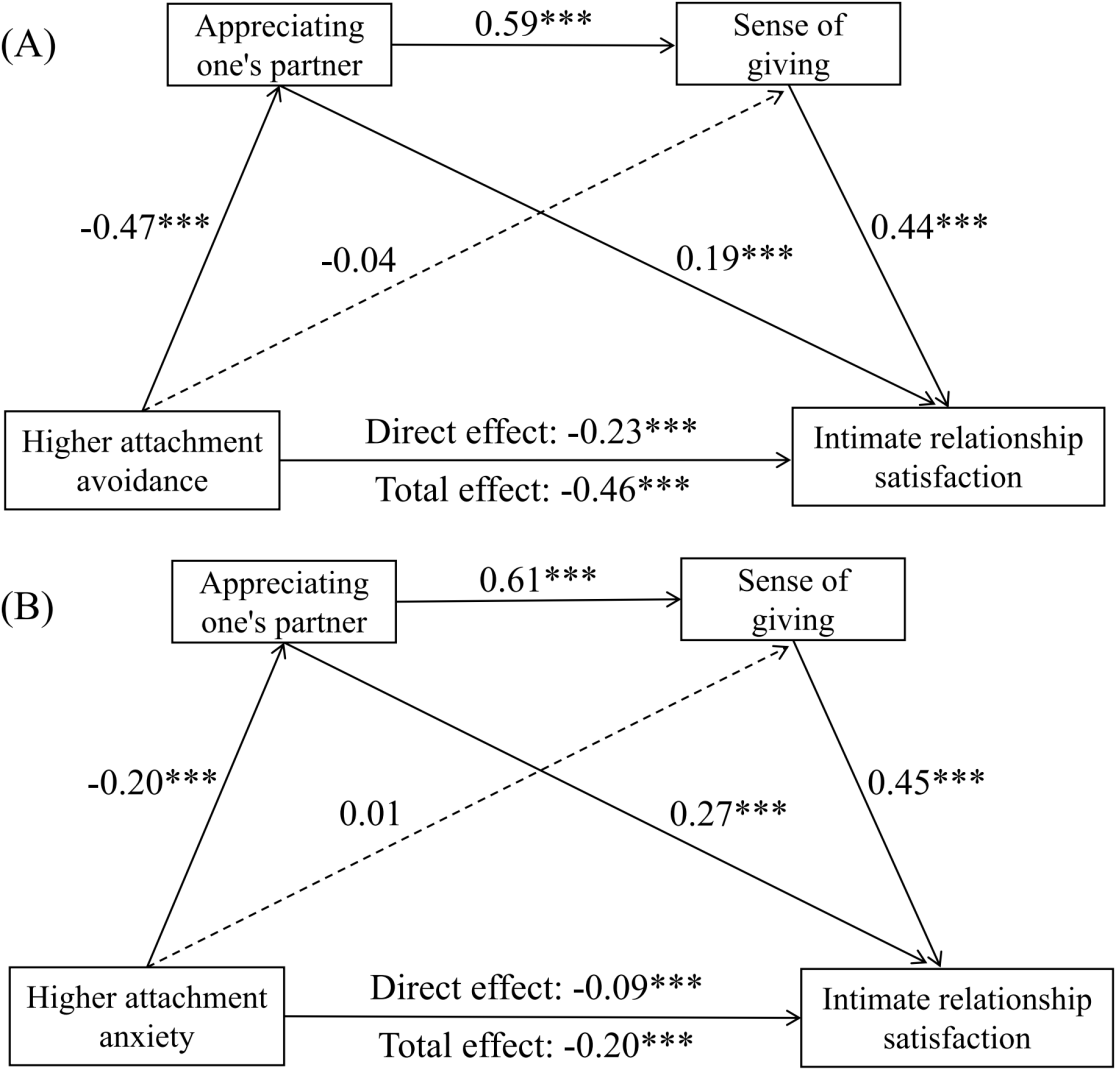
Chain mediation model depicting appreciating one’s partner and sense of giving as chain mediators of the relationship between adult attachment (avoidance **(A)** and anxiety **(B)**) and intimate relationship satisfaction. Solid lines indicate statistically significant results, while dashed lines indicate no statistical significance. ***p < 0.001.

Attachment Avoidance (**Figure 1A**). After controlling for covariates, higher attachment avoidance significantly and negatively predicted intimate relationship satisfaction (*β* = -0.23, *p* <.001) and appreciating one’s partner (*β* = -0.47, *p* <.001). However, higher attachment avoidance did not significantly predict sense of giving directly (*β* = -0.04, *p* >.05). Appreciating one’s partner, in turn, positively predicted both sense of giving (*β* = 0.59, *p* <.001) and intimate relationship satisfaction (*β* = 0.19, *p* <.001). Sense of giving also positively predicted intimate relationship satisfaction (*β* = 0.44, *p* <.001).

Attachment Anxiety (**Figure 1B**). Similarly, higher anxiety significantly and negatively predicted intimate relationship satisfaction (*β* = -0.09, *p* <.01) and appreciating one’s partner (*β* = -0.20, *p* <.001), but did not significantly predict sense of giving (*β* = 0.01, *p* >.05). The paths from appreciating one’s partner to sense of giving (*β* = 0.61, *p* <.001) and to intimate relationship satisfaction (*β* = 0.27, *p* <.001), and from sense of giving to intimate relationship satisfaction (*β* = 0.45, *p* <.001), were all significant.

Effect Decomposition. As detailed in **Table 2**; **Figure 1**, the total indirect effect for higher attachment avoidance was significant (effect = -0.32, 95% *CI* [-0.42, -0.22]), accounting for 50.00% of the total effect. The serial mediation pathway (Avoidance → Appreciating → Giving → Satisfaction) was also significant (effect = -0.17, 95% *CI* [-0.22, -0.14]). For higher attachment anxiety, the total indirect effect was significant (effect = -0.12, 95% *CI* [-0.19, -0.06]), accounting for 52.17% of the total effect, and the serial mediation pathway was also significant (effect = -0.07, 95% *CI* [-0.10, -0.03]). These results indicate that appreciating one’s partner partially mediated the associations between both attachment dimensions and intimate relationship satisfaction. Critically, the serial mediation pathway (attachment → appreciating one’s partner → sense of giving → intimate relationship satisfaction) was significant for both attachment avoidance and attachment anxiety.

**Table 2:** Chain mediation effects of appreciating one’s partner and sense of giving on the relationship between adult attachment and intimate relationship satisfaction.

Independent variable	Effect type	Path	Effect	Boot SE	95% *CI*	% of total
Higher attachment avoidance	Direct	AVO→SAT	-0.32	0.05	-0.41 ~ -0.22	50.00
	Indirect	AVO→APP→SAT	-0.13	0.04	-0.20 ~ -0.06	20.30
		AVO→GIV→SAT	-0.02	0.03	-0.08 ~ 0.03	3.13
		AVO→APP→GIV→SAT	-0.17	0.03	-0.22 ~ -0.14	25.57
	Total indirect effect		-0.32	0.05	-0.42 ~ -0.22	50.00
Higher attachment anxiety	Direct	ANX→SAT	-0.11	0.04	-0.18 ~ -0.04	47.83
	Indirect	ANX→APP→SAT	-0.06	0.18	-0.10 ~ -0.03	26.09
		ANX→GIV→SAT	0.01	0.02	-0.03 ~ 0.04	-4.35
		ANX→APP→GIV→SAT	-0.07	0.02	-0.10 ~ -0.03	30.43
	Total indirect effect		-0.12	0.03	-0.19 ~ -0.06	52.17

AVO, higher attachment avoidance; ANX , higher attachment anxiety; APP, appreciating one’s partner; GIV, sense of giving; SAT, intimate relationship satisfaction. *CI*, confidence interval. Confidence intervals that do not include zero indicate significant effects.

### 3.4 Chain mediation model 2: Feeling appreciated and sense of giving

A second chain mediation model was tested, replacing appreciating one’s partner with feeling appreciated by one’s partner as the first mediator.

**Figure 2:**
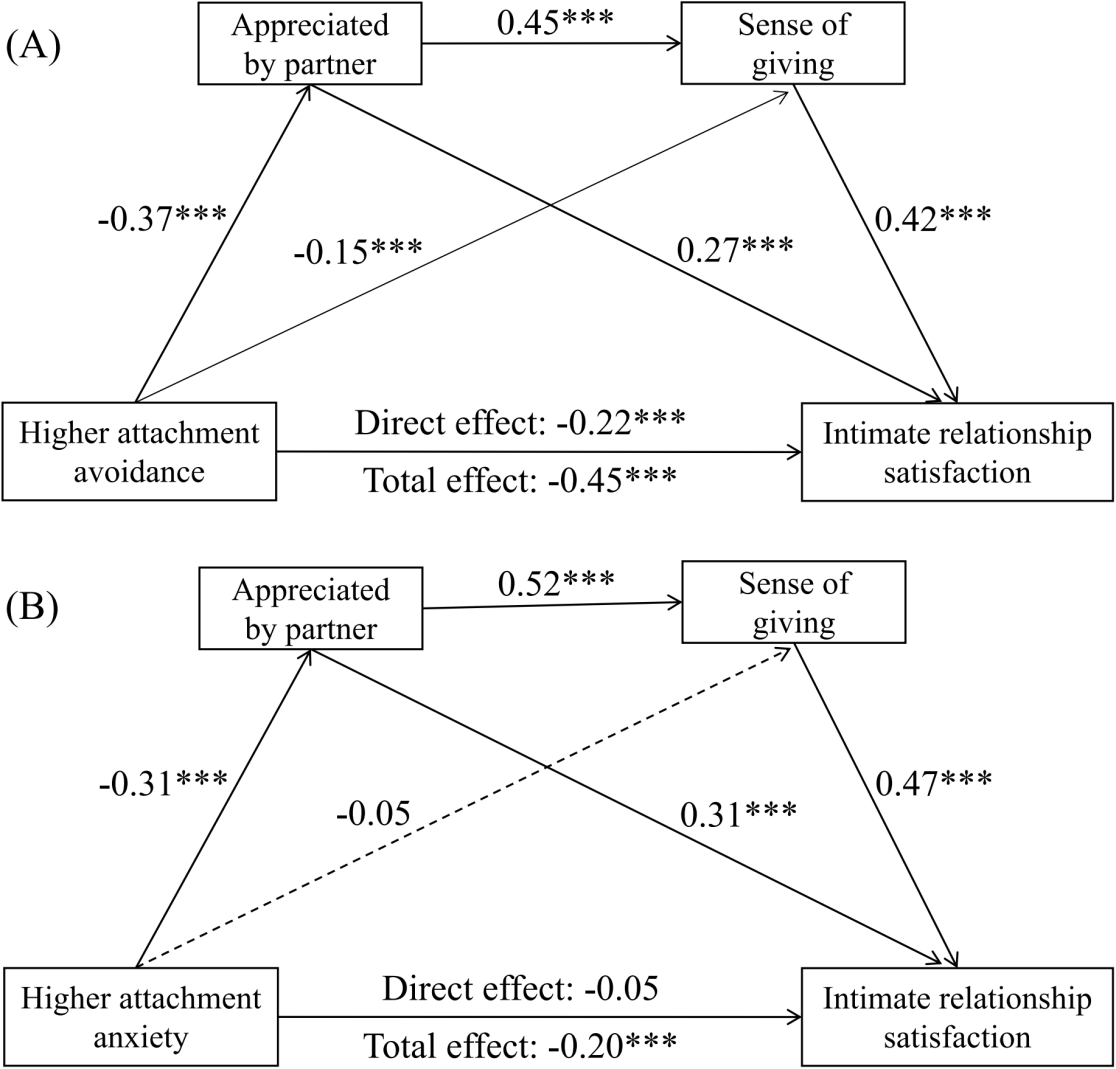
Chain mediation model depicting feeling appreciated by one’s partner and sense of giving as chain mediators of the relationship between adult attachment (avoidance **(A)** and anxiety **(B)**) and intimate relationship satisfaction. Solid lines indicate statistically significant results, while dashed lines indicate no statistical significance. ***p < 0.001.

Attachment Avoidance (**Figure 2A**). Higher attachment avoidance significantly and negatively predicted intimate relationship satisfaction (*β* = -0.22, *p* <.001), feeling appreciated by one’s partner (*β* = -0.37, *p* <.001), and sense of giving (*β* = -0.15, *p* <.001). Feeling appreciated positively predicted both sense of giving (*β* = 0.45, *p* <.001) and intimate relationship satisfaction (*β* = 0.27, *p* <.001). Sense of giving also positively predicted intimate relationship satisfaction (*β* = 0.42, *p* <.001).

Attachment Anxiety (**Figure 2B**). Higher attachment anxiety significantly and negatively predicted feeling appreciated by one’s partner (*β* = -0.31, *p* <.001) and relationship satisfaction (total effect *β* = -0.20, *p* <.001), but its direct path to sense of giving was not significant (*β* = -0.05, *p* >.05). Feeling appreciated positively predicted both sense of giving (*β* = 0.52, *p* <.001) and intimate relationship satisfaction (*β* = 0.31, *p* <.001). Sense of giving remained a significant positive predictor of intimate relationship satisfaction (*β* = 0.47, *p* <.001).

Effect Decomposition. The total effect of higher attachment avoidance on intimate relationship satisfaction was significant (effect = -0.64, *p* <.001), with a significant direct effect (effect = -0.31, 95% *CI* [-0.40, -0.23]) comprising 48.44% of the total effect. The total effect for higher attachment anxiety was also significant (effect = -0.23, *p* <.01). However, its direct effect on relationship satisfaction was not significant (effect = -0.06, 95% *CI* [-0.13, 0.01]), accounting for 26.09% of the total effect. This pattern of results suggests that the relationship between attachment anxiety and satisfaction is primarily mediated by the proposed indirect pathways. These findings support partial mediation: feeling appreciated partially mediated the association between both attachment dimensions and intimate relationship satisfaction, whereas sense of giving mediated this association only for higher attachment avoidance. Importantly, the serial mediation pathway (attachment → feeling appreciated → sense of giving → intimate relationship satisfaction) was significant for both higher attachment avoidance and attachment anxiety. Detailed results are presented in **Table 3**; **Figure 2**.

**Table 3:** Analysis of the chain mediation effect of appreciated by one’s partner and sense of giving in the relationship between adult attachment and intimate relationship satisfaction.

Independent variable	Effect type	Path	Effect	Boot SE	95% CI	% of total
Higher attachment avoidance	Direct	AVO→SAT	-0.31	0.05	-0.40 ~ -0.23	48.44
	Indirect	AVO→FAP→SAT	-0.14	0.03	-0.20 ~ -0.08	21.88
		AVO→GIV→SAT	-0.09	0.03	-0.15 ~ -0.03	14.06
		AVO →FAP→GIV→SAT	-0.10	0.02	-0.14 ~ -0.07	15.62
	Total indirect effect		-0.33	0.04	-0.40 ~ -0.25	51.56
Higher attachment anxiety	Direct	ANX→SAT	-0.06	0.04	-0.13 ~ 0.01	26.09
	Indirect	ANX→FAP→SAT	-0.11	0.03	-0.17 ~ -0.06	47.82
		ANX→GIV→SAT	0.03	0.02	-0.02 ~ 0.07	-13.04
		ANX→FAP→GIV→SAT	-0.09	0.02	-0.12 ~ -0.06	39.13
	Total indirect effect		-0.17	0.04	-0.24 ~ -0.10	73.91

AVO, higher attachment avoidance; ANX, higher attachment anxiety; FAP, feeling appreciated by partner; GIV, sense of giving; SAT, intimate relationship satisfaction. *CI*, confidence interval. Confidence intervals that do not include zero indicate significant effects.

## 4 Discussion

This study investigated the associations between adult attachment orientations and intimate relationship satisfaction in young adults, with a focus on potential mediating mechanisms involving appreciation and sense of giving. The results provided strong support for H1, H2, and H4. Hypothesis 3 received partial support: the single mediating role of sense of giving was significant only for higher attachment avoidance in the model involving feeling appreciated, but not for attachment anxiety or in the model involving appreciating one’s partner. Critically, the core chain mediation pathway (H4) was consistently significant across all models. In particular, the findings confirmed a chain mediation model, revealing that the negative association between insecure attachment (both higher avoidance and anxiety) and intimate relationship satisfaction is sequentially mediated by appreciation and sense of giving. This research not only confirms the foundational role of attachment in shaping relational outcomes but also illuminates a specific pathway through which these distal psychological working models translate into proximal relationship experiences. Notably, all primary findings remained robust after controlling for key demographic and relational variables (sex, age, only-child status, long-distance relationship status, and relationship duration), strengthening the credibility of our proposed model.

Consistent with a substantial body of prior research, our results reaffirmed that both higher attachment avoidance and attachment anxiety are significantly and negatively associated with intimate relationship satisfaction (63–67). Individuals with higher attachment avoidance often experience discomfort with intimacy and emotional distance, which is associated with lower relationship fulfillment. Likewise, those with higher attachment anxiety, who fear rejection and constantly seek validation, also tend to report less satisfaction in their partnerships. This finding aligns with core tenets of attachment theory, which posits that internal working models are related to perceptions and behaviors within close relationships, thereby contributing to their overall quality (68). Our study corroborates this foundational principle in a contemporary young adult population navigating a critical developmental stage for establishing long-term relational patterns.

A primary contribution of this study is the elucidation of the mediating roles of appreciation and sense of giving. Specifically, higher levels on both attachment dimensions (avoidance and anxiety) are associated with reduced expression of appreciation for a partner and a diminished feeling of being appreciated by them. For individuals with higher attachment avoidance, this deficit is likely linked to their deactivating strategies: expressing appreciation requires acknowledging dependence and emotional connection, which they actively suppress to maintain self-reliance (10). Similarly, their reduced feeling of being appreciated may arise from perceptual filtering: appreciative gestures from partners are downplayed or dismissed as threats to autonomy, further reinforcing emotional distance (48). For individuals with higher attachment anxiety, the deficit may be more perceptual. Their negative self-views and hypervigilance for signs of rejection may be associated with a tendency to discount, misinterpret, or fail to notice their partner’s appreciative gestures, thereby preventing them from feeling genuinely valued (69). In turn, their reduced expression of appreciation may reflect hyperactivating strategies: fear of non-reciprocation and abandonment inhibits proactive positive expressions, as such acts heighten vulnerability without assured return (70). This finding extends previous research on gratitude by identifying appreciation—the recognition of a partner’s intrinsic worth—as a key process that appears to be linked to insecure attachment, an association that may hinder the development of a secure and satisfying bond (71, 72).

Furthermore, this study advances a novel theoretical proposition by positioning sense of giving as a subsequent step in the mediational chain. The results suggest that the path from appreciation to sense of giving represents a critical link in the model. This finding supports the notion that feeling valued and appreciated is fundamentally linked to the subjective experience of investing in a relationship. Drawing on principles from social exchange and self-determination theories, feeling appreciated appears to reframe giving from a potentially burdensome ‘cost’ to a meaningful, autonomous expression of commitment (73, 74). When individuals feel their contributions are seen and valued, they experience giving as intrinsically rewarding and relationship-enhancing. Conversely, a lack of appreciation may be associated with perceiving such investments as unreciprocated and draining, potentially fostering resentment rather than satisfaction (75). The proposed model suggests a pattern in which insecure attachment is associated with lower relationship satisfaction through sequential associations with reduced appreciation and a diminished sense of giving.

The present findings accord with attachment theory and extend prior work on relational processes, yet require interpretation within the Chinese cultural context. Chinese norms emphasize interpersonal harmony, emotional restraint, and relational obligations, such that appreciation and giving in romantic relationships are predominantly expressed implicitly through instrumental acts, reciprocity, and maintenance of relational equilibrium, rather than explicit verbal or affective disclosure (76). Elevated attachment avoidance may further inhibit overt expressions, perceived as risking dependence and harmony (77). Heightened attachment anxiety may be intensified by indirect inference of emotional needs, diminishing perceived appreciation and giving tendencies (78). While core sequential mediation pathways remain robust in this young Chinese adult sample, mechanisms and effect sizes likely diverge in Western contexts valuing direct expressivity and autonomy (77, 79).

The significance of this research is multifaceted. Theoretically, it offers a more granular, process-oriented model that integrates attachment theory with concepts from social exchange theory and self-determination theory. By demonstrating a sequential mediational pathway linking attachment, appreciation, and sense of giving, we provide a clearer picture of how internal working models are related to dyadic functioning. From a public health perspective, these findings are particularly relevant given that relationship distress is a leading cause of mental health difficulties among young adults (75). Identifying malleable intervention targets is therefore crucial. Although attachment dimensions are relatively stable, behaviors and perceptions related to appreciation are more amenable to change. Our results suggest that interventions aimed at improving relationship quality may benefit from focusing on enhancing both the expression and perception of appreciation. Such efforts could potentially foster a positive reciprocal cycle of investment and satisfaction that may buffer against the negative associations of insecure attachment.

## 5 Limitations

Despite its contributions to understanding the mediating roles of appreciation and sense of giving in the relationship between adult attachment orientations and intimate relationship satisfaction among young Chinese adults, this study has several limitations.

First, the cross-sectional design precludes causal inferences. Although our proposed model is grounded in established theory, longitudinal research is necessary to confirm the temporal precedence of these variables. The cross-sectional design cannot empirically rule out alternative sequences; for instance, relationship satisfaction could foster a climate where appreciation and sense of giving are more likely to flourish. Future longitudinal studies using cross-lagged panel models are essential to test these alternative models.

Second, our reliance on self-report questionnaires may introduce CMV and social desirability bias. Although we conducted Harman’s single-factor test, which suggested that a single factor did not account for a substantial portion of the variance, this method is widely recognized as insufficient for conclusively ruling out CMV. Therefore, we cannot definitively exclude the possibility that CMV influenced the results, and interpretations should be made with appropriate caution. To more robustly address this concern, future research would benefit from incorporating stronger procedural remedies (such as temporal separation of measures) and alternative data collection approaches, including dyadic reports from both partners or observational methods, which can provide multi-source perspectives and reduce dependence on single-informant self-reports.

Third, the sample consisted exclusively of university students in China, which limits generalizability to other age groups (e.g., older adults) and non-student populations. Moreover, although the findings are presented in relation to young adults more broadly, cultural norms specific to the Chinese context—such as greater emphasis on emotional restraint, implicit expressions of appreciation and relational giving, and harmony-oriented interpersonal dynamics—may shape the observed associations in ways that differ from more individualistic cultures. This restricts cross-cultural generalizability, and future studies should examine these pathways across diverse cultural and age contexts to determine the extent to which the model is universal versus culturally specific.

Finally, certain psychometric properties warrant discussion. The attachment avoidance subscale demonstrated marginal internal consistency (*Cronbach’s α* = .69). However, supplementary confirmatory factor analysis indicated excellent model fit (*CFI* = .989, *TLI* = .966, *RMSEA* = .069), and composite reliability was.73, meeting standard thresholds. Additionally, we chose observed-variable path analysis over a full latent-variable structural equation model to maintain parsimony given our sample size. While this approach enhances comparability with prior literature, measurement error in observed variables may lead to underestimation of effect sizes. Future research using latent-variable models could yield more precise estimates.

## 6 Conclusion

This study elucidates a critical pathway through which adult attachment is associated with romantic relationship quality. The negative association between insecure attachment and intimate relationship satisfaction is sequentially mediated by appreciation and sense of giving. Specifically, insecure attachment is linked to a reduced expression and perception of appreciation. This, in turn, is associated with diminished intrinsic rewards from relational giving, which is ultimately linked to lower satisfaction. These findings underscore the importance of appreciation as a pivotal mechanism in relational functioning and suggest that public health interventions designed to improve young adults’ mental health may benefit from targeting appreciation to encourage a more positive cycle of relational giving and satisfaction.

## Data Availability

The raw data supporting the conclusions of this article will be made available by the authors, without undue reservation.

## Supplementary material

Supplementary Figure 1 Correlation heatmap of study variables.
