# Supplementary figures and images for: Adult attachment and intimate relationship satisfaction among university students: the chain mediating roles of appreciation and sense of giving
Source: Front Psychiatry. 2026 Apr 24;17:1758775. doi: 10.3389/fpsyt.2026.1758775 (PMC13153132; doi:10.3389/fpsyt.2026.1758775)

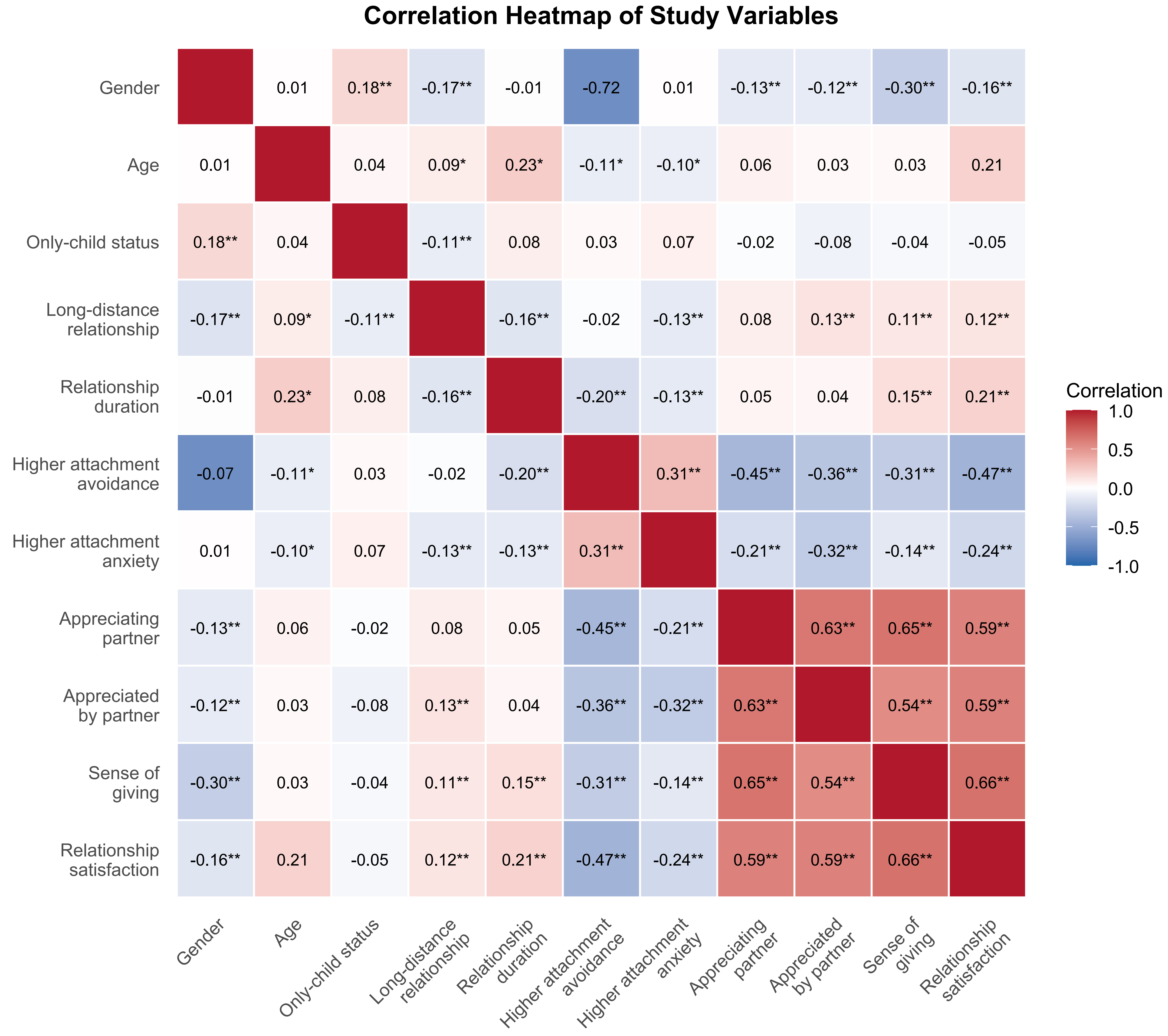

Supplement: Supplementary Figure 1 — Correlation heatmap of study variables. [file Image1.tif]
